# Energy system contribution during competitive cross-country skiing

**DOI:** 10.1007/s00421-019-04158-x

**Published:** 2019-05-10

**Authors:** Thomas Losnegard

**Affiliations:** Department of Physical Performance, Norwegian School of Sport Sciences, Ullevål Stadion, Post box 4014, 0806 Oslo, Norway

**Keywords:** Anaerobic capacity, Cross-country skiers, Elite athletes, Maximal aerobic power, Oxygen deficit, Pacing pattern

## Abstract

Energy system contribution during cross-country (XC) skiing races is dependent on several factors, including the race duration, track profile, and sub-techniques applied, and their subsequent effects on the use of the upper and lower body. This review provides a scientific synopsis of the interactions of energy system contributions from a physiological, technical, and tactical perspective. On average, the aerobic proportion of the total energy expended during XC skiing competitions is comparable to the values for other sports with similar racing times. However, during both sprint (≤ 1.8 km) and distance races (≥ 10 and 15 km, women and men, respectively) a high aerobic turnover interacts with subsequent periods of very high work rates at ~ 120 to 160% of *V*O_2peak_ during the uphill sections of the race. The repeated intensity fluctuations are possible due to the nature of skiing, which involves intermittent downhills where skiers can recover. Thus, the combination of high and sustained aerobic energy turnover and repeated work rates above *V*O_2peak_, interspersed with short recovery periods, distinguishes XC skiing from most other endurance sports. The substantially increased average speed in races over recent decades, frequent competitions in mass starts and sprints, and the greater importance of short periods at high speeds in various sub-techniques, have demanded changes in the physiological, technical, and tactical abilities needed to achieve world-class level within the specific disciplines.

## Introduction

**Fig. 1:**
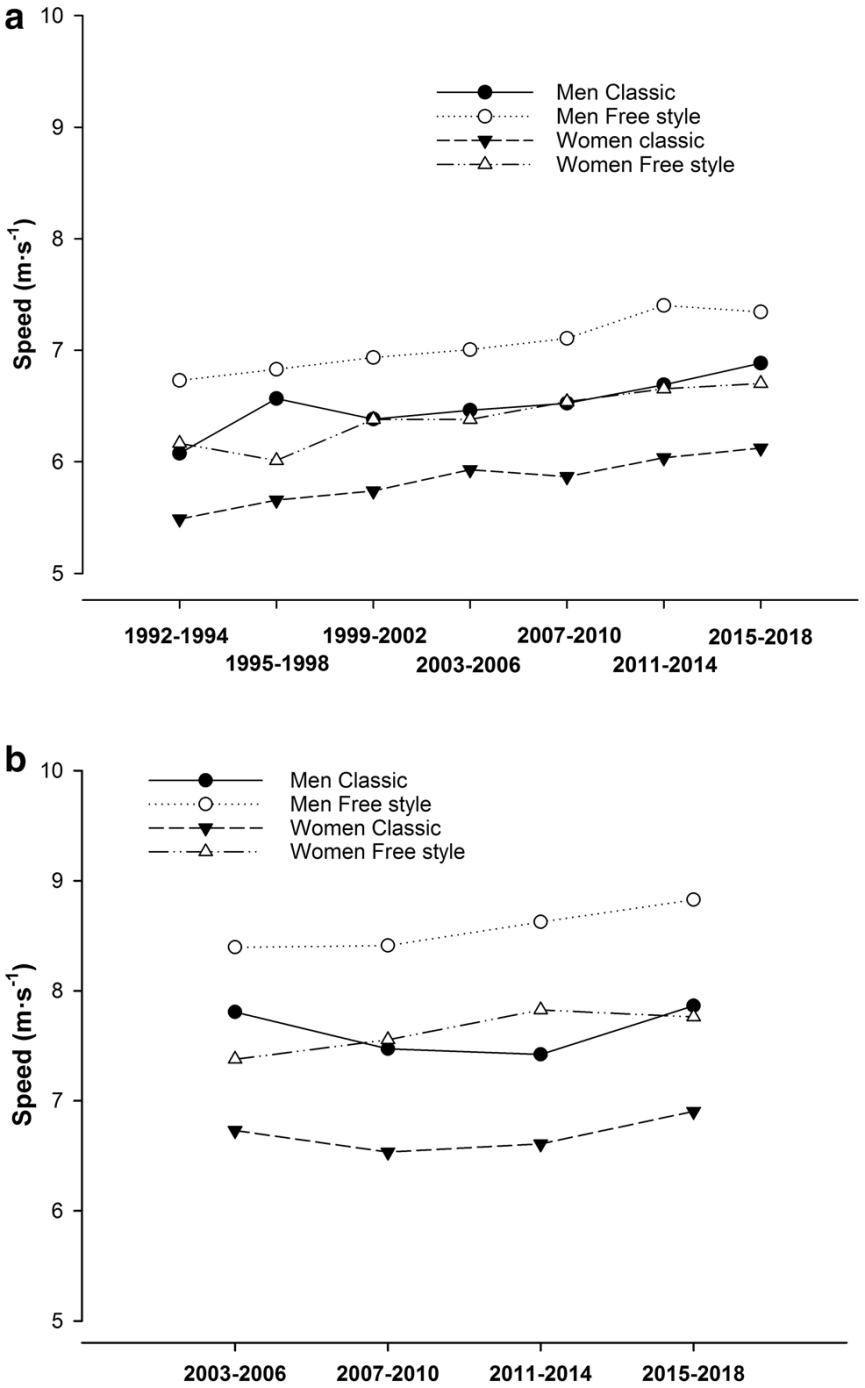
Average speed during **a** distance (10 and 15 km, women and men, respectively) and **b** sprint prologue (time-trial) over an Olympic cycle. The year represents the season, e.g., races in November–December 2002 are taken as season 2003. All data are from world cup, World championship and Olympic races. Distance and sprint races are taken as the average speed for the three best skiers in each race (sprint: number of free style races = 88, classic races = 73, distance; number of free style races = 60, classic races men/women = 74). Average speed is calculated as 10 or 15 km divided by time in distance skiing and exact distance in meters divided by time in sprint. All data from individual starts, e.g., prologue during sprint events. Data collected are based on final results from FIS (2018)

Nearly 100 years ago, the winner of the traditional ski race, Vasaloppet, took 7.5 h to complete the 90 km race. Since then, remarkable changes have occurred in cross-country (XC) skiing, including improved equipment, track preparation, technique, and training strategies. This has permitted skiers to reduce their metabolic cost by more than 50% per meter (Formenti et al. 2005), allowing today’s elite skiers to finish the race in well under 4 h. Such an increase in average speed has also been evident in recent decades, exemplified by a ~ 10% speed increase in international elite distance races from 1992 to 2018 (≥ 10 and 15 km, women and men, respectively). Moreover, with the introduction of “head-to-head” sprint skiing in the late 1990s (competition distance < 1.8 km, ~ 3 min), ~ 11 to 17% higher average speeds in sprint compared to distance skiing are evident, implying different performance abilities compared to distance skiing (Fig. 1). Further, as 5 out of 6 events in the Olympics today are mass starts, the final outcome is often decided by the ability to accelerate rapidly during the race and/or in the end-spurt to break away from the group.

**Fig. 2:**
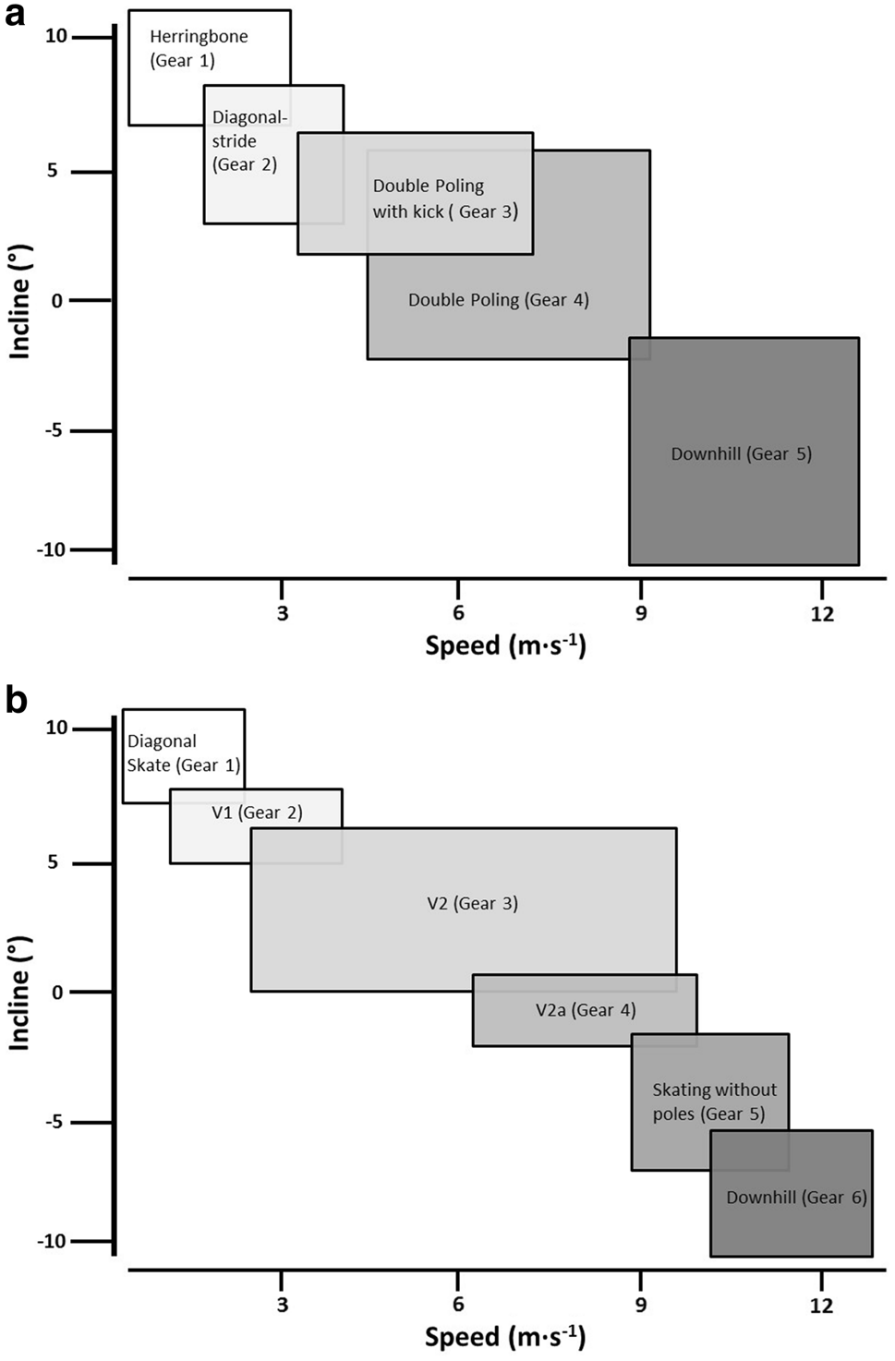
Illustration of the gear-system in classic (**a**) and free style (**b**). Based on Andersson et al. (2010, 2014, 2016), Gløersen et al. (2018), Losnegard et al. (2012b), Marsland et al. (2017) and Pellegrini et al. (2013) and unpublished data. **a** Herringbone (Gear 1); arm and leg movement in a diagonal fashion without ski gliding before leg kick. Ski slightly angled. Diagonal stride (Gear 2); arm and leg movement in a diagonal and paralell fashion before leg kick. Double poling with kick (Gear 3); symmetrical poling action while ski gliding followed by one leg kick, double poling (Gear 4); symmetrical poling action while ski gliding without leg kick. Downhill (Gear 5); downhill tuck position without pole- and leg actions. **b** Diagonal skate (Gear 1); arm and leg movement in a diagonal fashion. Technique involving one-pole action for each leg stroke. V1 (Gear 2); asymmetrical double poling action during every two leg strokes. V2 (Gear 3); parallel poling action for every leg stroke. V2a (alternate skate) (Gear 4); parallel poling action for every two leg strokes. Skating without poles (G5); only leg strokes are performed without poling. Downhill (G6); downhill tuck position without pole- and leg actions

**Fig. 3:**
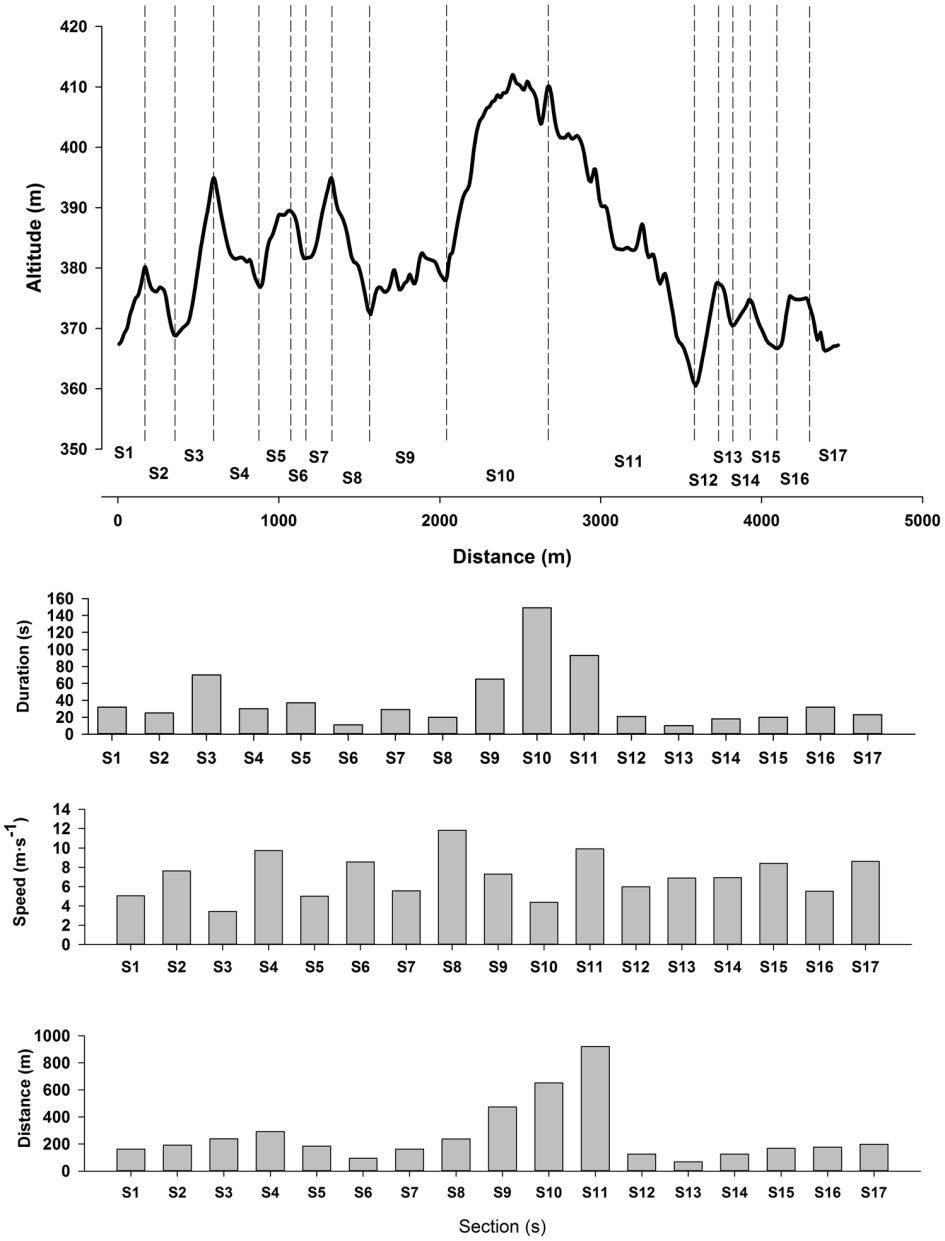
Race profile and distance of selected segments in the 4.6 km international course at Holmenkollen, Oslo, Norway coupled with duration and speed in each segment performed by male elite cross-country skiers during a simulated 3 × 4.6 km race on rollerski Modified based on data from Karlsson et al. (2018)

The international XC skiing race program consists of two separate techniques (classic style and free style; called ski-skating) each with several sub-techniques. The choice of sub-technique depends mainly on speed and therefore acts as a gearing system (Andersson et al. 2010; Losnegard et al. 2012b; Marsland et al. 2017; Nilsson et al. 2004a; Pellegrini et al. 2013) (Fig. 2a, b). Unlike most other endurance sports, a substantial variation in speed exists during races, since competition courses in XC skiing consist of approximately one-third ascending, one-third flat and one-third descending terrain (FIS 2017). In general, about 50% of the total time is used in uphill skiing, while the remaining ~ 35 and 15% is spent in flat and downhill sections. For instance, a distance race of 10–15 km lasts about ~ 26 to 35 min, implying that ~ 13 to 18 min are spent in uphill (~ 50%) and 6–8 min (~ 25%) in flat sections. However, the duration of any specific segment (uphill, flat or downhill) is typically ~ 10 to 35 s and rarely above 70 s in an international race course (Fig. 3). Recent data also indicate the presence of a hierarchy in the contribution of time spent on uphill, flat, and downhill terrains to overall time-trial performance (Bolger et al. 2015; Sandbakk et al. 2011a, b; Andersson et al. 2010). Thus, the uphill sections are the most discriminating sections during races, although better skiers generally perform better in all types of terrain. Given the large variation in speed, and thus incline and turns, skiers normally perform about 25 transitions between sub-techniques per km (Marsland et al. 2017; Sandbakk et al. 2016b; Solli et al. 2018; Andersson et al. 2010). These factors are special aspects of XC skiing and clearly demand both efficient skiing techniques and efficient transitions between sub-techniques. Therefore, the ability to master a wide range of sub-techniques is crucial to overall performance.

Sport-specific peak aerobic power (*V*O_2peak_) is one of the main determinants of performance in sprint and distance XC skiing (Losnegard and Hallen 2014b). In addition, because of sudden and repeated changes in metabolic requirements, the capability to sustain rapid changes in metabolic energy turnover during a race is an important factor for high-level skiers. The anaerobic turnover rate has mainly been investigated during sprint events. These competitions involve large accelerations, implying a periodically very high metabolic power output (Andersson et al. 2017; Losnegard et al. 2012a; Sandbakk et al. 2011a). However, recent data collected in field conditions using advanced wearable sensor technology underline the importance of being able to repeatedly perform at metabolic rates well above *V*O_2peak_ in distance skiing as well (Gløersen et al. 2018, 2019; Karlsson et al. 2018). Hence, similar to XC skiing, a significant part of the race during mountain bike cross-country Olympic format is performed above maximal aerobic power (Hays et al. 2018). The nature of these sports is clearly different to other endurance sports such as running or swimming in terms of energy turnover during races. Accordingly, the “traditionally” held view that the anaerobic energy system plays an insignificant role during distance skiing events seems to warrant re-evaluation.

The purpose of the present review is first to provide a scientific synopsis of the energy system contributions from the closely related aspects of physiology, technique, and tactics in competitive XC skiing. Second, we discuss the importance of aerobic power and anaerobic capacity in determining performance in sprint versus distance events. Third, the methodology used to investigate aerobic and anaerobic power/capacity in XC skiing is discussed in light of the large variations in terrain and thus in speed and technique.

### Energy system contributions in endurance sports

XC skiing is an endurance sport in which the average speed for a required distance (distance time^−1^) is mainly determined by the metabolic energy turnover maintained by the athlete throughout the race (energy time^−1^) divided by the economy of progression, or energy cost of locomotion (energy distance^−1^) (di Prampero 2003; di Prampero et al. 1986). Accordingly, a greater metabolic energy turnover and/or a reduced cost of locomotion will increase speed and thus improve performance. Total metabolic power results from the contributions of the three main metabolic energy-yielding pathways that are closely integrated to match the energy requirement imposed by the muscles. While the anaerobic energy-yielding system is supported by the splitting of high-energy phosphates from PCr and the substrate-level phosphorylation of anaerobic glycolysis, the aerobic system refers to oxidative phosphorylation of ATP through the oxidation of carbohydrates and fats (Gastin 2001). In general, the anaerobic systems can produce high metabolic power, but they have limited energy capacities. In contrast, the aerobic system has a large capacity, but is characterized by a considerably lower maximal power and a considerable inertia. The relative contributions of the energy-yielding pathways are highly dependent on the race duration, meaning that there are precise sport-specific demands (Gastin 2001; Spencer and Gastin 2001). However, as the following section will demonstrate, the energy system contributions during XC skiing races are not only dependent on the duration of the event, but also on the course profile and thus the pacing pattern. Moreover, with varying terrains and therefore varying speeds, different sub-techniques are employed, using the upper and lower body to different extents, which seems to influence the energy system contribution.

### Energetics during XC skiing competitions

#### Distance skiing

Three decades ago, pioneering work by Norman et al. (1989) and Norman and Komi (1987) proposed, based on biomechanical calculations, that elite skiers had a mechanical power output on uphill sections of 600–700 W, corresponding to 100–120% of estimated *V*O_2peak_. These early findings implied that a significant anaerobic energy turnover occurred within races, even though this was observed in a relatively long race event (up to ~ 1 h 30 min, 30 km race). Later, Mygind et al. (1994) examined energy metabolism during a simulated classic and freestyle race (13.8 km, 42–50 min) on snow. Here, O_2_-uptake was determined using Douglas bags on flat and uphill sections, in addition to measurements of blood lactate concentration ([La^–^]_b_) during the race. As the *V*O_2_ was ~ 90% of *V*O_2peak_ in both terrain sections and with both techniques, and since these terrain sections consisted of more than 85% of the total race time, a very high aerobic turnover was evident during the race. Moreover, the [La^–^]_b_ was found to be constantly high (~ 10 mmol L^−1^) during the simulated race, which supported the earlier findings that a high anaerobic turnover rate occurs during parts of such races. Welde et al. (2003) confirmed these observations using a portable breath-by-breath system to analyze *V*O_2_ responses during a simulated race in both the classic and ski-skating technique in well-trained junior female skiers. During a 6 km (~ 22 min) ski race on snow, similar O_2_-uptakes for both techniques were found (average of ~ 84%), with the highest value corresponding to 94% of *V*O_2peak_ obtained during the race. These data are similar to those presented by Mognoni et al. (2001) (90–95% of *V*O_2peak_), based on calculations from heart rate (HR) profiles in well-trained skiers during a race.

**Fig. 4:**
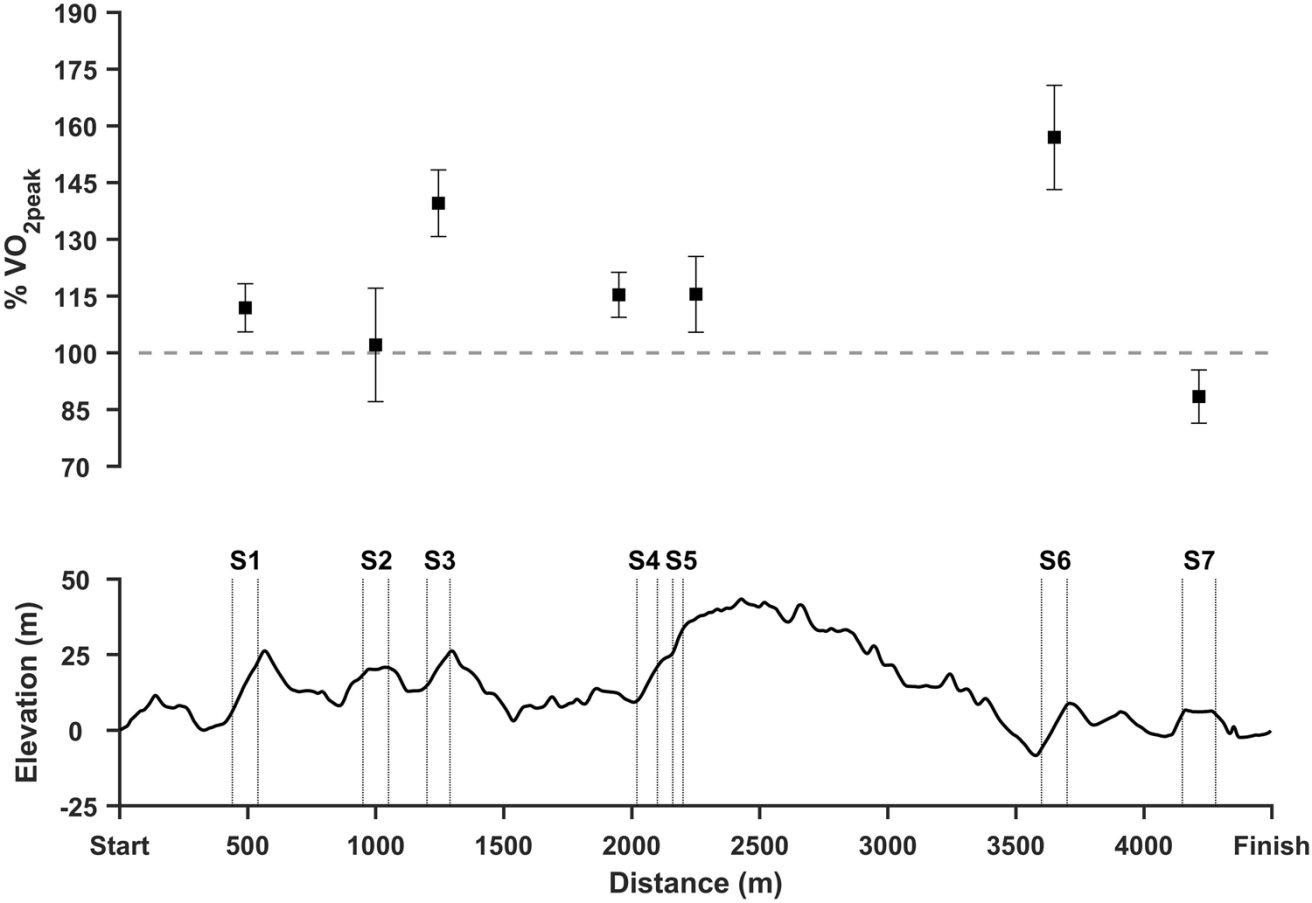
Race profile and calculated exercise intensity (O_2_-demand expressed as  % of *V*O_2peak_) during different segments (flat and uphill) of a 15 km simulated free style race performed by well-trained skiers Modified based on data from Karlsson et al. (2018)

To enhance the understanding of how the different energy sources contribute to the overall energy output during distance races, we conducted a series of studies using wearable sensor-based methods (Gløersen et al. 2018, 2019; Karlsson et al. 2018). These studies combined kinematic field measurements during roller skiing with laboratory tests on a treadmill to estimate the total energy turnover in races. In a simulated 15-km time-trial race (~ 33 min), the *V*O_2_-demand in well-trained skiers (peak aerobic power; *V*O_2peak_; 72 mL kg^−1^ min^−1^) was found to frequently exceed their *V*O_2peak_ with work rates ranging from 100 to 160% of *V*O_2peak_ (Karlsson et al. 2018) (Fig. 4). In a subsequent study, an outdoor roller ski race (15-km) performed by elite skiers (*V*O_2peak_; 78 mL kg^−1^ min^−1^) was reproduced indoors on a treadmill to measure the O_2_-uptake under controlled conditions (Gløersen et al. 2019). The main findings were that skiers acquired small to moderate oxygen deficits during individual uphill segments, ranging from 0 to 50% of each athlete’s maximal accumulated oxygen deficit (ΣO_2_-deficit). These data suggest that the maximal ΣO_2_-deficit per se is not a major determinant of distance skiing performance; rather, it is the ability to repeatedly perform workloads above *V*O_2peak_. Moreover, Gløersen et al. (2019) confirmed earlier findings that elite skiers have a high aerobic turnover during the race (~ 90 to 95% of *V*O_2peak_), but that the *V*O_2peak_ is not reached during the race. The reason for this is not clear; however, a possible explanation is the relationship between work rate and duration of the specific sections. The duration of the longest uphill segment (~ 120 s) was sufficient to elicit *V*O_2peak_; however, athletes chose to reduce their work rate to ~ 100% (or slightly less) of *V*O_2peak_ in this segment. This contrasts with work rates in the shorter uphill segments, which were substantially higher than *V*O_2peak_ (~ 15 s; ~ 160% of *V*O_2peak_). Consequently, skiers lowered their work rate in longer uphill sections, probably in an attempt to reduce fatigue from the perspective of the remaining race time (Gløersen et al. 2018, 2019; Karlsson et al. 2018).

#### Sprint skiing

With the introduction of sprint skiing to the World Championships in 2001 and the Olympics in 2002, a large body of scientific evidence on the physiological demands has emerged. Stoggl et al. (2007) constructed a simulated race indoors on a roller ski treadmill and found *V*O_2_ values corresponding to ~ 95% of *V*O_2peak_, implying a high aerobic turnover during the ~ 3 min race. In addition, Vesterinen et al. (2009) and Stoggl et al. (2007) showed high peak ([La^–^]_b_) after simulated races (> 12 mmol L^−1^). Although blood lactate concentration is only a rough estimate of anaerobic turnover, these findings also implied that the anaerobic contribution to the total energy yield is substantial in sprint XC skiing, but notably, no clear differences were found compared to distance skiing (Mygind et al. 1994). Therefore, to give a more detailed description of the relative contributions of various energy sources in sprint skiing, Losnegard et al. (2012a) used the ΣO_2_-deficit method in XC skiing. This concept was introduced by Krogh and Lindhard (1920) and later re-evaluated by Medbo et al. (1988) in running, where supramaximal energy production was estimated by extrapolating the linear relationship between external load and the steady-state O_2_ cost at submaximal intensities. Losnegard et al. (2012a) found that the distribution of aerobic versus anaerobic energy supply was ~ 75/25% during a ~ 3 min test, as in events of similar duration in other sports (Gastin 2001). These findings were later confirmed in several other studies on roller skiing (Losnegard et al. 2013, 2015; Andersson et al. 2016, 2017; McGawley and Holmberg 2014; Moxnes and Moxnes 2014).

In terms of exercise intensity, skiers produce supramaximal power outputs, well above their *V*O_2peak_ (110–120% of *V*O_2peak_), during simulated races of ~ 3 to 4 min at constant incline (Losnegard et al. 2012a, 2013, 2015). Interestingly, these supramaximal power outputs during a single time-trial were found to be more similar to those relating to 800 m (113%) than to 1500 m (103%) running in highly trained athletes (Losnegard et al. 2012a; Spencer and Gastin 2001). This is of practical importance, as sprint skiing is traditionally related to 1500-m running performance because of the duration of the races. However, exercise intensity is not only related to duration, but also depends on factors such as the volume of muscle mass recruited, which is greater in skiing because of the use of all four limbs for progression; the activation pattern; and the altimetry of the courses (Hermansen and Saltin 1969; Olesen 1992; Bojsen-Moller et al. 2010). Sandbakk et al. (2011a) estimated the work rate during an uphill section (270 m, ~ 51 s) in a sprint competition on snow in elite skiers and found that the energy demand was ~ 160% of *V*O_2peak_. Later, Andersson et al. (2017) found that the total metabolic power corresponded to 120–130% of *V*O_2peak_ during the uphill sections of simulated indoor roller skiing sprint time trials, where the course consisted of only uphill and flat terrain. Moreover, Andersson et al. (2019) found that the metabolic power was similar for both sexes during a simulated race outdoor on rollerski (~ 130% of *V*O_2peak_). These estimates from “long” uphill sections in sprints (~ 1.8 km) are not very different from the data for distance skiing (15 km) (Gløersen et al. 2019; Karlsson et al. 2018), despite the significantly shorter total duration (~ 3 vs 35 min). This implies that the very high work rates observed in uphill segments are not necessarily dependent on total race duration but more likely on the course profile and pacing pattern, as later discussed.

Several authors have addressed the importance of anaerobic capacity in time-trial sprint performance in recent years. Losnegard et al. (2012a) showed that in groups of male sprint, distance and long-distance skiers, anaerobic capacity was the single most important factor for the time-trial sprint performance on a treadmill. Likewise, it has been demonstrated that specialized elite sprint skiers have a significantly higher anaerobic capacity, directly related to higher body mass, than distance skiers (Losnegard and Hallen 2014b). These studies suggested that a clear specialization had occurred in XC skiing, at least for males. For female skiers, no such clear specialization is evident, based on the average top-ten world cup standings in sprint and distance skiing. Over the five seasons from 2013 to 2018, 3.2 ± 1.8 female skiers (mean ± SD) were ranked on both lists per season, in contrast to males, where 1.6 ± 0.9 skiers appeared on both lists (FIS 2018). This difference is probably related to the fact that female skiers in world cup races demonstrate a substantially greater variability between subjects than men, implying that the standard of competition is greater for male than for female skiers (Spencer et al. 2014).

Overall sprint skiing performance is also related to the ability to maintain subsequent performance over four heats with recovery periods of ~ 60–40–20 min (prologue–quart–semi-final) (Andersson et al. 2016; Haugnes et al. 2018; Losnegard et al. 2015; Mikkola et al. 2010). Andersson et al. (2016) found that the within-subject variation in performance from heat to heat was primarily explained by the ability to reproduce the maximal ΣO_2_-deficit. Here, the two fastest trials were associated with substantially larger anaerobic energy supply, while the oxygen uptake during trials was similar. The reason why some skiers are able to recover the ΣO_2_-deficit faster than others is not fully understood. Losnegard et al. (2015) found that skiers with the largest rate of disappearance of [La^–^]_b_ performed better compared with skiers with the lowest rate of disappearance of [La^–^]_b_ after active recovery. Furthermore, the relative change in rate of disappearance of [La^−^]_b_ was highly related to the ability to sustain a maximal ΣO_2_-deficit in subsequent heats. Björklund et al. (2011) used a rollerski treadmill with alternating exercise intensity of 70 and 90% of *V*O_2peak_ in a group of elite and well-trained skiers. They found that the elite skiers had a larger rate of disappearance of [La^–^]_b_ in the transition from exercise intensity at 90–70% of *V*O_2peak_ than the well-trained skiers. They therefore suggested that this ability is an important factor for performance in XC skiing (Björklund et al. 2011). Similar findings were obtained by (Sandbakk et al. 2011b), who concluded that world class sprint skiers have a faster rate of disappearance of [La^−^]_b_ than national-level skiers. However, the importance of the rate of disappearance of [La^−^]_b_ is debated (Gladden 2004), and its relationship to subsequent performance should be interpreted with caution.

The ability to reproduce a high ΣO_2_-deficit in subsequent heats is also dependent on the recovery time. A reduction in ΣO_2_-deficit between two heats with a recovery time of 22 min between heats has been reported, whereas no difference was found with 42 min of recovery (Losnegard et al. 2012a, 2015). This notion is important from a practical perspective, since skiers who compete in the first semifinal have a slightly longer recovery time than skiers in the second semifinal and thereby have a potential performance benefit.

Despite the increasing number of studies focusing on the importance of anaerobic capacity in skiers, only a few studies are available that focus on female skiers. In general, when groups of men and women with similar training backgrounds have been examined in other sports, the maximal ΣO_2_-deficit in women is typically 10–30% lower than in men (Medbo and Burgers 1990; Weber and Schneider 2000, 2002). In XC skiers, McGawley and Holmberg (2014) found a “likely practical difference” (47 vs 41 mL kg^−1^) in maximal ΣO_2_-deficit between young males and females, respectively (~ 17 years). In senior athletes, world-class female skiers seem to have a substantially lower maximal ΣO_2_-deficit (~ 40%) than their male counterparts (Tables 1, 2) and this difference is significantly higher than the O_2_-cost or *V*O_2max_ relative to body mass (Sandbakk et al. 2014). The sex differences in performance seem also to increase with increased upper-body work (such as V2 skating and double poling compared to diagonal stride), which could, at least in part, be related to the higher lean body mass in the upper body in males compared to females (Hegge et al. 2016; Sandbakk et al. 2014). Taken together, this could imply that the observed greater sex differences in performance using the V2 skate or double poling techniques compared to the diagonal stride may be related to muscle mass in the upper body and thus the ability to achieve a high ΣO_2_-deficit.

### Energetics and pacing pattern during cross-country skiing

It is widely recognized that athletes’ ability to distribute their work and energy expenditure throughout the race, described as their “pacing strategy” or “pacing pattern”, has a significant impact on performance. Due to the varying inclines, skiers continuously change speed, making the description of pacing patterns complex in XC skiing. Several studies have shown that elite skiers adopt a positive pacing pattern (reduced velocity) on a lap-to-lap basis in both sprint and distance races (Andersson et al. 2010; Bolger et al. 2015; Formenti et al. 2015; Karlsson et al. 2018; Larsson and Henriksson-Larsen 2005; Losnegard et al. 2016; Welde et al. 2017). However, the large variations in exercise intensity during races (e.g., uphill vs downhill) and thus the disassociation between exercise intensity and speed in different terrains means that describing pacing patterns exclusively in terms of inter-lap variations in speed is insufficient (Abbiss and Laursen 2008; Karlsson et al. 2018). Moreover, a direct assessment or estimation of the energy turnover is difficult because of the unknown anaerobic energy contribution to the overall energy turnover and the non-steady-state metabolic power imposed by tactics and varying terrain. Therefore, several authors have suggested the use of external power in various terrains as a reasonable proxy for the total metabolic power output and the way these metabolic pathways may contribute during races (Gløersen et al. 2018; Karlsson et al. 2018; Moxnes et al. 2014; Sundström et al. 2013; Swaren and Eriksson 2017; Moxnes and Moxnes 2014; Sandbakk and Holmberg 2017). The consensus of these studies is that the power output is higher during uphill than flat skiing and that considerable variations in power output and metabolic demand occur during a race.

Norman and Komi (1987) calculated the metabolic cost of skiing on level (1.6°) and uphill terrain (9°) during the 15-km World championships in Lahti in 1978, at a time when skiers used the classic diagonal stride technique in both conditions. They found that the metabolic cost during uphill sections was substantially higher than for level terrain (154 vs 60 mL kg^−1^ min^−1^). These findings were also confirmed using skating techniques 30 years later, although the metabolic cost in flat terrains turned out to be higher (100–110 of *V*O_2peak_) than previously shown, probably due to the evolution of flat-terrain techniques (Gløersen et al. 2019; Karlsson et al. 2018). Thus, studies show that skiers, in addition to positive pacing on a lap-to-lap basis, demonstrate a variable pacing pattern according to terrain and speed in an attempt to optimize performance (Andersson et al. 2010, 2016; Gløersen et al. 2018; Karlsson et al. 2018; Norman and Komi 1987; Sundström et al. 2013; Swaren and Eriksson 2017).

**Fig. 5:**
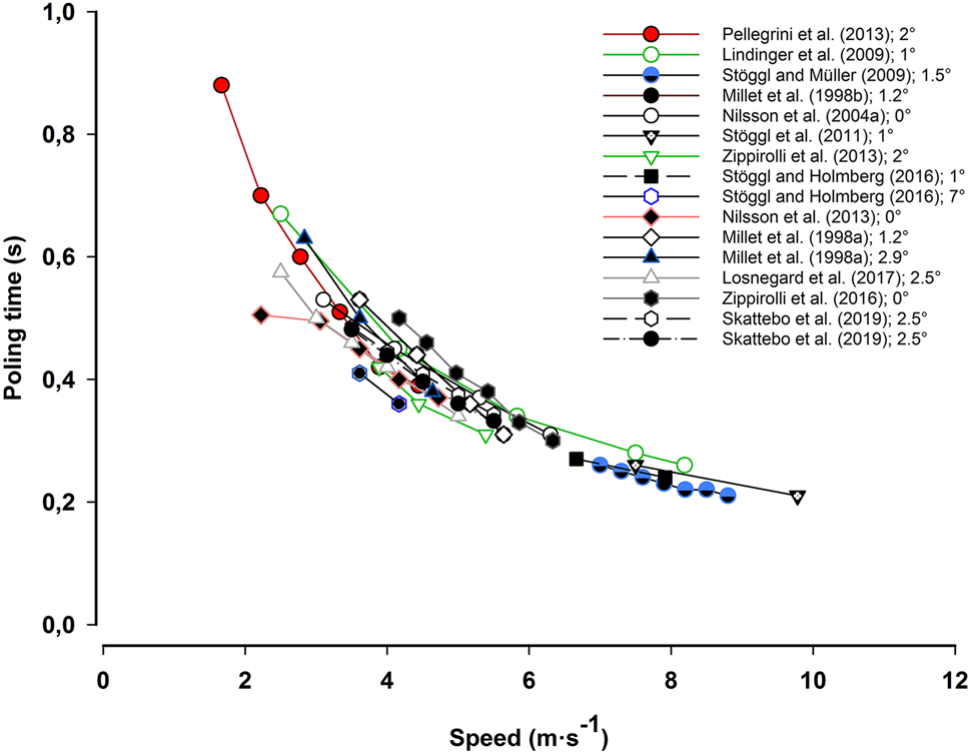
Relationship between speed and poling time in double poling. Numbers beside references in the figure show the incline at which the test was conducted. Data taken from Millet et al. (1998a, b), Nilsson et al. (2004a, 2013), Lindinger et al. (2009), Stoggl and Muller (2009), Stoggl et al. (2011), Stoggl and Holmberg (2016), Pellegrini et al. (2013), Losnegard et al. (2017), Zoppirolli et al. (2013, 2016) and Skattebo et al. (2019)

The rationale for applying higher power outputs and intensities in uphill vs flat sections of a race may involve several reasons. First, at high speeds (flat terrain), a large fraction of the increased power is dissipated to overcome air drag. In contrast, at lower speeds and during uphill skiing, this fraction is negligible. Secondly, a higher ΣO_2_-deficit is found in diagonal stride than double poling (Andersson et al. 2017), which, together with higher *V*O_2peak_, could imply that the total available energy turnover is lower during double poling than in diagonal stride for elite skiers. These factors are likely to influence the pacing pattern over different terrain sections due to different maximal energy turnover rates for different sub-techniques (e.g., double pooling versus diagonal stride). Third, as shown in running (Olesen 1992) and XC skiing (Karlsson et al. 2018), the maximal ΣO_2_-deficit is greater during uphill than flat sections, possibly due to a greater muscle mass being involved and/or biomechanically limiting factors at high speeds. In contrast to uphill skiing, a short pole contact time is the main limiting factor during flat skiing. As shown in (Fig. 5), speeds above 7 m s^−1^, independent of incline and ski friction, result in poling times below 0.3 s. This somehow prevents skiers from gaining sufficient force-impulse to further increase their speed and, therefore, increase their exercise intensity. The differences in maximal ΣO_2_-deficit between sub-techniques (e.g., diagonal stride vs double poling), tested at different speeds, could therefore be related to pole contact time as a limiting factor for further increases in metabolic rate. The short contact time at high speeds may also limit the ability to accumulate a high ΣO_2_-deficit within specific techniques, confirmed by the findings from ski skating (Karlsson et al. 2018). Moreover, unpublished data from our lab indicate that the maximal ΣO_2_-deficit in double poling is lower on flat terrain compared to uphill terrain in elite skiers, although *V*O_2peak_ is similar in both terrains. The speeds in this study corresponding to an oxygen demand of 70 mL kg^−1^ min^−1^ during flat (1°) and uphill (8°) sections are ~ 8 m s^−1^ and ~ 2.4 m s^−1^, respectively. This example shows that the short contact time is a major factor that needs to be taken into consideration. Further, it indicates that the lower exercise intensity observed during XC ski racing on flat vs uphill terrain is intrinsically limited by the biomechanics of skiing at various speeds (Andersson et al. 2017; Karlsson et al. 2018; Norman and Komi 1987). Therefore, individual technical abilities may strongly affect the total energy turnover rates and thus the pacing patterns of elite skiers on various terrains.

### Energetics of various techniques

Several studies have demonstrated that male and female world-class skiers are among the endurance athletes with the highest *V*O_2max_. Accordingly, world-class performance has been associated with maximal values above 70 and 80 mL kg^−1^ min^−1^ or 4.0 and 6.0 L min^−1^, in female and male skiers, respectively (Ingjer 1991; Saltin and Astrand 1967; Sandbakk et al. 2016a; Holmberg et al. 2007; Tonnessen et al. 2014; Losnegard and Hallen 2014b; Losnegard et al. 2013) (Tables 1, 2). However, due to the substantial variation in speed during a competition, skiers must have the ability to master a wide range of sub-techniques not only from a technical but also from a physiological perspective. In XC skiing, the work is shared between the arms, trunk, and legs, and the contribution of each depends on the movement in specific sub-techniques and the relative intensity generated by the skier (Bojsen-Moller et al. 2010; Calbet et al. 2004, 2005; Danielsen et al. 2015; Rud et al. 2014; Zoppirolli et al. 2017). Hence, the ability to produce high aerobic power while performing various sub-techniques seems crucial for performance (Sandbakk et al. 2016b).

**Table 1:** Anthropometry, peak aerobic power and maximal accumulated oxygen deficit (ΣO_2_-deficit) in world-class, -elite and national-level distance male skiers

Variables	World-class skiers (*n *= 5)	Elite skiers (*n* = 5)	National skiers (*n* = 5)
Height (cm)	180 ± 6	178 ± 6	183 ± 2
Body-mass (kg)	74 ± 7	71 ± 3	76 ± 4
Body-mass index (kg m^2^)	22.6 ± 0.7	22.2 ± 1.2	22.8 ± 1.3
FIS-distance points	2 ± 1	28 ± 6	74 ± 16
*V*O_2peak_ (mL kg^−1^ min^−1^)	82 ± 3	81 ± 6	76 ± 3
*V*O_2peak_ (L min^−1^)	6.1 ± 0.6	5.7 ± 0.3	5.8 ± 0.5
ΣO_2_-deficit (mL kg^−1^)	76 ± 11	75 ± 11	76 ± 18

Values are mean ± SD. *Significant differences between groups*, P* < 0.05. All data collected in the V2 technique during rollerski treadmill testing from October to February. From Losnegard et al. (2012a, b, 2013), Losnegard and Hallén (2014a, b) and unpublished

**Table 2:** Anthropometry, maximal aerobic power and maximal accumulated oxygen deficit (ΣO_2_-deficit) in world-class and national-level distance female skiers

Variables	World-class skiers (*n *= 6)	National skiers (*n* = 6)
Height (cm)	165 ± 4**	170 ± 3
Body mass (kg)	60.0 ± 5.1	62.6 ± 5.8
Body mass index (kg m^2^)	22.0 ± 1.1	21.7 ± 1.5
FIS-distance points	8 ± 10*	72 ± 22
*V*O_2peak_ (mL kg^−1^ min^−1^)	71 ± 3*	65 ± 4
*V*O_2peak_ (L min^−1^)	4.2 ± 0.2*	4.0 ± 0.4
ΣO_2_-deficit (mL kg^−1^)	47 ± 8	48 ± 11

Values are mean ± SD. *Significant differences between groups*, P* < 0.05. All data collected in the diagonal stride technique during rollerski treadmill testing from October to February. Data from Sandbakk et al. (2016a)

The individual *V*O_2peak_ seems to follow an “inverted U-shape curve” depending on the relative contributions from the upper and lower body (Bergh et al. 1976; Losnegard and Hallen 2014a). It has been shown that if the arms contribute 10–30% of the total work rate, whole body *V*O_2max_ increases, while at more than 30% of the total power output, whole body *V*O_2max_ decreases (Bergh et al. 1976). This is of special importance in XC skiing with various sub-techniques depending on different muscle use in the upper and lower body. During the diagonal stride, studies have found that skiers exhibit ~ 3% higher *V*O_2peak_ than in running (Holmberg et al. 2007; Stromme et al. 1977; Welde et al. 2003). Pellegrini et al. (2011) found that in the diagonal stride, the ratio between power exerted from the arms and total power output decreases with increasing incline. Moreover, Calbet et al. (2004) found that blood flow and O_2_-uptake in the legs increased to a greater extent than in the arms when intensity increased. In other skiing techniques, *V*O_2peak_ is typically ~ 3% lower in ski skating and ~ 10% lower in double poling than in running (Losnegard and Hallen 2014a; Losnegard et al. 2014; Rundell 1996). Here, the relative contribution of total power output from the arms seems higher than in the diagonal stride (Millet et al. 1998a, b; Rud et al. 2014). Taken together these findings can help explain why skiers seem to have a higher *V*O_2peak_ in the diagonal stride than in running, whereas *V*O_2peak_ in double poling and ski skating is lower.

**Fig. 6:**
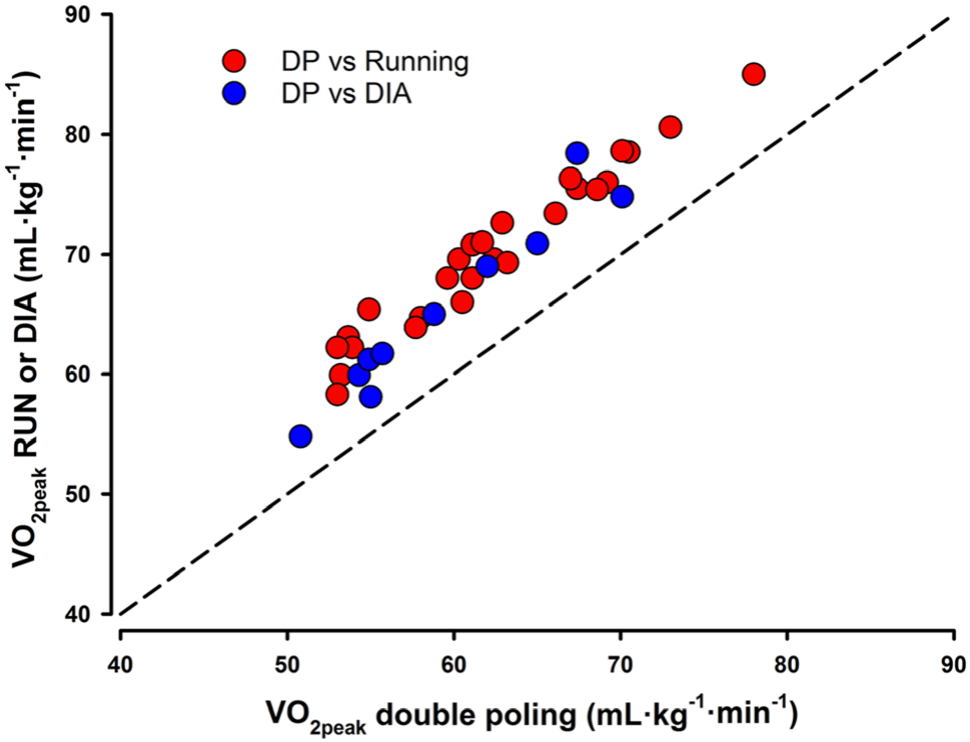
Relationship between *V*O_2peak_ tested in double poling and *V*O_2peak_ tested in running (RUN) or diagonal stride (DIA). Line of identity is shown as a dashed line. From Sandbakk et al. (2014, 2016a), Skattebo et al. (2016, 2019), Hegge et al. (2015), Fabre et al. (2010), Vandbakk et al. (2017), Holmberg and Nilsson (2008), Borve et al. (2017), Carlsson et al. (2014, 2016), Bucher et al. (2018), Sagelv et al. (2018), Holmberg et al. (2007), Bojsen-Moller et al. (2010), Losnegard et al. (2014), Bjorklund et al. (2015), Nilsson et al. (2004b), Rud et al. (2014), Stadheim et al. (2013, 2014) and Hoff et al. (2002)

For senior elite skiers who already have a high *V*O_2peak_, further increases in *V*O_2peak_ seem difficult (Losnegard et al. 2013). Therefore, reducing the “gap” between *V*O_2peak_ in a specific exercise mode and *V*O_2peak_ in running seems like an appropriate training strategy. These aspects have received attention in recent decades, particularly because of the greater use of double poling in various terrains. In an invasive state-of-the-art experiment, Calbet et al. (2005) demonstrated that elite XC skiers have similar O_2_-extraction in the arms to that found in physically active subjects in the legs, but still lower than in the leg muscles of well-trained subjects. The authors proposed that this is due to the lower mean capillary transit time and smaller diffusing area in the arms compared to the legs (Calbet et al. 2005). Further, Rud et al. (2014) tested well-trained XC skiers in double poling (*V*O_2peak_; ~ 61 mL kg^−1^ min^−1^) at low and moderate intensity and showed that the increased O_2_-uptake in the arms (~ 20%) was due to increased blood flow, while the increased O_2_-uptake in the legs (~ 50%) was related to both increased blood flow and O_2_-extraction. Moreover, as the exercise intensity increases, the arms have a net release of lactate, while the legs have a net lactate uptake during double poling (Rud et al. 2014; Van Hall et al. 2003). These studies thus indicated that the arms have a lower muscular oxidative capacity than the legs even in well-trained XC skiers. However, how training-induced changes can influence the maximal O_2_-uptake in double poling is not fully understood, since no studies have been able to demonstrate via experimental data that upper-body endurance training leads to an increased *V*O_2peak_ in double poling relative to *V*O_2peak_ in running. Reviewing studies that have compared *V*O_2peak_ in double poling to *V*O_2peak_ in running or diagonal stride have shown that *V*O_2peak_ during double poling is in average 12% (range 5–18%) lower than during running or diagonal stride. In general, these data imply that the ratio is independent of sex (12% for men, 11% for women) or *V*O_2peak_ in running (Fig. 6). Moreover, this relationship seems independent of level or specialization. This was recently addressed in a comparison study between elite long-distance and distance skiers (Skattebo et al. 2019). Although the best long-distance skiers are highly specialized in the use of double poling in training and competitions, the *V*O_2peak_ double poling/*V*O_2peak_ running was not different to the distance skiers (10% vs 9%) or lower-level skiers in other studies. These findings seem consistent with a similar study, testing *V*O_2peak_ in double poling and diagonal stride in long-distance and distance skiers (Sagelv et al. 2018). Interestingly, in a recent study by Ortenblad et al. (2018), the mitochondrial volume percentage and the number of capillaries per fiber area were similar in the arms and legs, although more type-2 fibers were evident in the arms for well-trained skiers. Moreover, Terzis et al. (2006) found that extensive upper-body training induced similar adaptions in the triceps brachii as seen in legs after 20 weeks of double poling training. Here, the number of capillaries around each fiber increased as well as enzyme activities and muscle fiber size. These studies indicate that the upper-body muscles of today’s elite skiers could exhibit similar adaptations to those seen in the leg muscles. Therefore, it could be suggested that the consistently lower *V*O_2peak_ found in double poling or ski skating compared to running might be related to other factors than the oxidative capacity in arm muscles, which should be investigated in future studies.

In addition to investigating muscle use and maximal aerobic power between sub-techniques, changes in muscle activation with increasing intensity within specific sub-techniques have received attention over the last decade (Bojsen-Moller et al. 2010; Danielsen et al. 2015; Rud et al. 2014; Zoppirolli et al. 2017). Such information is important when optimizing the specificity of physiological and technical aspects during training for elite skiers. This has been of particular interest in double poling, where propulsive forces through the poles increase with increased intensity/speed. Importantly, this does not imply the development of higher metabolic rates by the upper body at skiing speeds. In fact, during double poling the relative contribution from the legs increases with increasing work intensity, since the arm and shoulder muscles reach a plateau in energy turnover. Further increases in whole body exercise intensity during double poling are covered by muscles in the legs (Bojsen-Moller et al. 2010; Danielsen et al. 2015; Rud et al. 2014; Zoppirolli et al. 2017; Holmberg et al. 2005, 2006), reflected by a greater vertical displacement of the center of mass. Thus, increases in power output create changes in movement patterns and thus in muscle use. These aspects are clearly demanding from a training perspective, as they challenge competitive skiers to impose sufficient training loads on specific muscles performing specific movement patterns in the various techniques.

### Methodological considerations in determining the energetics during XC skiing

In any tests the observed value will differ from the true value due to measurement errors. The two most important aspects of measurement error are concurrent validity and retest reliability (Hopkins 2000). In general, the validity and reliability for systems to measure aerobic energy systems under steady-state conditions in the lab is well documented (Foss and Hallen 2005). However, in recent decades portable open-circuit spirometry systems have been more commonly used in sports science, with the advantage of enabling field-based measurement. Although several studies have evaluated their reliability and validity (Macfarlane and Wong 2012; Overstreet et al. 2017; Perez-Suarez et al. 2018; Vogler et al. 2010) under controlled conditions, little data are available for these systems in cold environments. Moreover, few studies have evaluated these systems at the high ventilation rates (> 200 L min^−1^) normally seen in elite male skiers (Losnegard and Hallen 2014b; Skattebo et al. 2019).

Validity studies of maximal ΣO_2_-deficit in general are difficult to conduct due to the lack of a comparable “gold standard” method for whole-body exercise. Some studies have used anaerobic ATP production from muscle biopsy data to investigate the validity of ΣO_2_-deficit (Bangsbo et al. 1990), but since the active muscle mass is unknown in whole-body exercises, such methods have several disadvantages (Noordhof et al. 2010). Moreover, limited information is available on the reliability of estimating ΣO_2_-deficit during XC skiing. To quantify reliability in any test, change in the mean, re-test correlation, and within-subject variation appears to be the most important measurements (Hopkins 2000). When a subject is tested several times, a random variation between trials occurs, seen in the standard deviation of the individual values. Such within-subject variation in also referred to as the typical error of measurement. Losnegard et al. (2013) reported a typical error (as the coefficient of variation; CV) in ΣO_2_-deficit during roller ski skating of 8.1%, which is substantially higher than the errors in *V*O_2peak_ (2.3%), O_2_-cost (1.2%) and 1000-m performance (2.7%) on the treadmill. Similar findings were made by Losnegard et al. (2015) with CVs of 9.8, 3.6, and 3.6% for ΣO_2_-deficit, *V*O_2peak_ and 800-m performance, respectively. Hence, based on limited available data, the typical error of the ΣO_2_-deficit method in roller ski skating seems large, and this must be taken into consideration when interpreting results.

To date, studies in XC skiing have exclusively used one-to-six submaximal workloads each with durations of 4–6 min, but the validity of such method for XC skiing is limited. In the original paper by Medbo et al. (1988), the authors proposed that a minimum of 8–10 measurements of the steady state O_2_-uptake, taken as the O_2_-uptake during a period of 8–10 min of exercise at constant intensity, is needed. This method has been questioned by others as the duration on the submaximal workloads seem to influence the measured *V*O_2_-values (Bangsbo 1992). Whipp and Wasserman (1972) showed at high exercise intensity that the *V*O_2_ continued to increase after 3 min and did not reach steady-state values after 6 min. This indicates that the slow component of the *V*O_2_-kinetics influences the constructions of the O_2_-demand (Ozyener et al. 2003). These aspects were reviewed thoroughly by Noordhof et al. (2010) and general limitations of the maximal ΣO_2_-deficit are provided here.

In XC skiing, anaerobic capacity has been estimated using both the maximal ΣO_2_-deficit method (Losnegard et al. 2015, 2012a, 2013; Losnegard and Hallen 2014b; McGawley and Holmberg 2014; Sandbakk et al. 2016a) and the gross-efficiency method (Andersson et al. 2016, 2017). In general, these methods have shown a high level of disagreement, implying that they should not be used interchangeably (Andersson and McGawley 2018; Noordhof et al. 2010). However, except for the recent comparison study of different methods by Andersson and McGawley (2018), no study has systematically compared different estimates of anaerobic capacity in XC skiing, and further studies are clearly warranted.

The combination of varying contributions from upper and lower body, changes in incline/speed ratio, and thus movement pattern within a sub-technique, potentially influences the ratio of workload to metabolic rate and thereby affects the ΣO_2_ deficit. Andersson et al. (2017) stated that the same incline should be used on both the submaximal and supramaximal workloads to avoid changes in the O_2_-cost per watt or gross efficiency. This statement is based on a large body of evidence that a substantial increase in incline will influence O_2_-cost per watt in both classic and ski-skating styles (Andersson et al. 2017; Karlsson et al. 2018; Sandbakk et al. 2013). However, it seems plausible that the movement pattern and the different contribution from upper and lower body are largely dependent on both speed and incline. This is exemplified in double poling, where at increasing speed or incline, skiers demonstrate a substantial change in movement pattern with more use of the legs to cover the increased metabolic demand (Bojsen-Moller et al. 2010; Rud et al. 2014; Sandbakk et al. 2016a; Stoggl and Holmberg 2016; Zoppirolli et al. 2017). Hence, more detailed data on how speed and incline affect O_2_-costs in different skiing techniques are needed to optimize methods for estimating anaerobic capacity.

## Perspectives and further research questions

It is well established that a very high maximal aerobic power, coupled with high efficiency, is essential for achieving world-class level in XC skiing. In recent decades, these aspects have been analyzed in detail for various techniques over a large range of inclines and speeds, due to the development of skiing-specific treadmills and ergometers. Moreover, due to the altered competition formats influencing the tactical, biomechanical, and physiological demands during events, a growing interest in the fluctuating contributions of aerobic and anaerobic energy sources during competition has emerged. However, much remains to be done in this field.

The unique, repeated supramaximal workload demands in sprint and distance skiing require both a technique-specific high aerobic energy turnover and the ability to recover the oxygen deficits within short periods of time. An important but unsolved question is how to optimize the recovery of the energy reserve during intermittent exercise, which is possible whenever the O_2_-demand is lower than the “critical power” (Chidnok et al. 2012). The large variations in exercise intensity during XC skiing races (e.g., uphill vs downhill) suggest that this is an important factor for performance. Much of the literature from a physiological perspective in XC skiing has focused on constant intensity protocols on rollerski treadmills, and hence, investigating the intermitted exercise in XC skiing “in real life competitions” is warranted. Of special note is the *V*O_2_-kinetics, that is likely to play an important role for exercise tolerance in XC skiing. A fast *V*O_2_-kinetics at the onset of exercise will reduce the O_2_-deficit needed to obtain the O_2_-demand (Davies et al. 2017), which during a competition with repeated supramaximal workloads could be of great importance. Moreover, the duration of any specific segment (uphill, flat or downhill) is typically ~ 10 to 35 s in an international race course (Fig. 3). During such short intermitted exercise, the contribution from substrate-level phosphorylation to the ATP production has shown to be of great importance in knee-extensor exercises (Davies et al. 2017). Here, future studies combining intra-muscular bioenergetics with kinematical models in a sport-specific content may further enhance our understanding about the demands of XC skiing. Although new insight has recently been provided (Gejl et al. 2017), much remains to be solved in this field.

Exercise intensity during a competition is highly related to the pacing pattern exhibited by the skier. Although it is well accepted that skiers demonstrate a positive pacing pattern on a lap-to-lap basis and that a variable pacing pattern occurs within laps, no experimental data are available to establish what constitutes the “optimal” pacing pattern in XC skiing. In particular, information is lacking on mass-start races on the importance of a high work intensity during the closing part of such races, which makes it vital to conserve power for the final spurt. Moreover, researchers must take into account the different speeds, terrains, altitudes, temperatures and surface conditions, and how they interact with the different sub-techniques where work is shared between the upper and lower body to different extents. The growing knowledge of computer simulations and enhanced methods to estimate external power in various terrains, combined with improved technology, means that new possibilities in this area are clearly developing.

The hyperbolic relationship between exercise duration and exercise intensity is a well-known aspect in endurance sports. This “critical power concept”, is an alternative method to investigate the energetic demands in sports, which has been widely applied also to intermittent exercise (Jones and Vanhatalo 2017; Poole et al. 2016). The critical power (or speed) concept seems as a promising tool in XC skiing as it integrates the aerobic and anaerobic contributions to the performance. However, except for the recent study by Gløersen et al. (2019), no study has used this concept in XC skiing. Future studies should therefore validate this approach for XC skiing, based on the recent critical power model applicable to intermittent exercise (Skiba et al. 2012, 2014).

Finally, over the recent years, a growing interest in Paralympic skiing has emerged. Here, the large individual variations in energy systems contributions, muscle use and competitions formats clearly demands alternative methods for testing and training (Baumgart et al. 2018; Bhambhani et al. 2012) which should be investigated more in the future.

## Summary and conclusion

A non-steady work rate is a distinct aspect of competitive XC skiing that is unlike most other endurance sports. In XC skiing, high demands are placed on the interaction of aerobic and anaerobic systems during competitive sprint and distance skiing. Moreover, the maximal ΣO_2_-deficit, combined with higher body mass, seems an important factor for discriminating specialized sprint skiers from distance skiers. In addition, both sprint and distance skiing demand the ability to reproduce and recover O_2_-deficits within races and/or between heats in sprint skiing. Thus, within sprint or distance skiers’ specializations, it may not be the maximal ΣO_2_-deficit per se that is crucial to the overall performance, but rather the ability to repeat high-intensity periods with a high rate of recovery. A high rate of recovery from ΣO_2_-deficit is also important for producing a variable pacing pattern, where elite skiers frequently exceed their *V*O_2peak_ in uphill sections with work rates as high as 120–160% of *V*O_2peak_.

The various sub-techniques demand different contributions from the upper and lower body muscles, which change with increasing intensity. Although a specific scientific emphasis has been to enhance the peak aerobic power in various sub-techniques to the same level as in running, no studies have shown convincing direct experimental data. Thus, the effects of increased upper-body training on performance seem to be related not to the *V*O_2peak_, but rather to other factors such as the fractional utilization, work economy/efficiency or anaerobic capacity.

Finally, the complexity of XC skiing is also challenging from a testing perspective to ensure control of reliability and validity in various testing situations, and specifically during outdoor tests on snow. Of special note is the lack of information on methods for estimating anaerobic capacity in XC skiing. Such information is important for future studies to increase the knowledge of energy system contributions in XC skiing.

## Abbreviations

ATP: Adenosine triphosphate
CV: Coefficient of variation
HR: Heart rate
[La^–^]_b_: Blood lactate concentration
O_2_-cost: Oxygen cost
PCr: Creatine phosphate
*V*O_2max_: Maximal oxygen uptake or maximal aerobic power
*V*O_2peak_: Peak oxygen uptake or peak aerobic power
W: Watt
XC: Cross-country
ΣO_2_-deficit: Accumulated oxygen deficit
